# Supplementary material for: The effect of face masks on confusion of emotional expressions
Source: PLoS One. 2025 Sep 10;20(9):e0330430. doi: 10.1371/journal.pone.0330430 (PMC12422498; doi:10.1371/journal.pone.0330430)
Supplement: Attachment — Submitted filename: LibbyAndScarince_R&R_Response.docx [file pone.0330430.s002.docx]

**Response to Reviewers**

Due to the substantial changes to the paper, we have summarized the major changes by section below and address specific actions to comments by the reviewers following the overall summary (highlighted in yellow).

**General Summary**

Introduction (literature review and theoretical framing)

- We heavily restructured the introduction to focus on consistent finding from before and after the COVID-19 pandemic. We do still emphasis Carbon’s (2020 & 2022) work as the motivation and method for our experiments were heavily based on it, but we further emphasized how our work builds on previous findings.
- We clarified the use of basic emotional theory (and, subsequently, the facial action coding system in the discussion) as the main theoretical formulation for our experiments. To help emphasis this, we introduce and discuss action units in the introduction as the central aspect of how occlusion affects accuracy.
- Along with the restricting of the introduction, we incorporated more recent findings in our literature review. We incorporated more recent work related to face coverings post-pandemic and targeted the review of emotional intelligence to its role in interpreting emotional expressions.

Experiment 1 (method and results)

- We provided more clarity in the explanation for why we limited view time of the images as a way to increase the difficulty of the recognition task to investigate the effect of emotional intelligence.
- Based on feedback from Reviewer 2, we reanalyzed our results with target emotion as a within-subjects factor and included additional figures to accompany those results. We also provided clarification for using the separate regressions to investigate the interaction between the time targets were visible with participant emotional intelligence.
- To reduce repetition between the individual experiment results sections, discussion sections, and the general discussion, we combined the results are discussion for the individual experiments. This includes more interpretation along the presentation of the results.

Experiment 2 (method and results)

- We provided more explanation for the changes made in Experiment 2 based on the limitations of the method and findings of Experiment 1.
- We also implemented the same structural changes made to Experiment 1 in Experiment 2, including the additional factor of emotion expression in our analysis.

General Discussion

- As with the introduction, we also made substantial structural changes to the general discussion. Most of the discussion is still centered on interpreting recognition errors in the context of obscured and shared action units, but we relate more of our findings to other recent findings, particularly when discussing confusion rates.
- Additionally, we further discussed the mixed findings regarding emotional intelligence.

**Reviewer 1**

The article investigates whether wearing facial masks has effects on emotion recognition accuracy, and whether emotion intelligence and timing of the expression seen mediate the effects. The authors conducted two studies to exam the effects. The findings from the articles were interesting, and experiments were well designed. However, the writing, organizations and technical soundness needs to be significantly improved. I do not recommend the article to be accepted in its current form. I will discuss my comments below

1. The introduction is poorly written. This is my biggest concern with the article.
a. First of all, the discussion of emotion theory, facial mimicry, emotion intelligence, emotion expression do not connect with each other at all. All I see is a pile of short summaries of research papers, and they do not connect to the arguments. All the descriptions do not connect to your hypothesis

Action: We made substantial changes to the structure and content of the introduction. In terms of flow, we give a brief overview of the using action units to identify expressions and pre-pandemic basic research on the consequences of obscuring action units. We then review research on emotion recognition in response to the COVID-19 pandemic. This review is more comprehensive than our previous submission, but we do still emphasize Carbon’s work, as it was the primary basis for our set of experiments. We then briefly review research about individual differences in emotion recognition, including the possible role of emotional intelligence. This leads into the current investigation, where we are more explicit about the selection of our variables compared to the prior research we reviewed.

b. Discussion of emotion theory is not accurate. For example, page 3. Basic emotion theory states that there are some basic emotions that are universally recognized. They reflect individual’s internal states, originated by survival instincts and biological needs. The authors mixed the concept of basic emotion theory and functional emotional theory. This is not acceptable. Page 4., authors claims that mouth region relays more emotional information that eye region. This is also not accurate.

Action: Thank you for pointing that out. We have clarified our overview to stay within the scope of basic emotion theory. We also contextualized the conclusion from Blais et al. (2012) about the diagnostic value of the mouth region to be less definitive.

c. There are many more articles studying similar topics since covid-19, and the authors failed to recognize them. For example, when you are talking about facial regions, facial masks, and emotion recognition, especially when you cited Dimberg’s work on facial mimicry out of nowhere, this immediately reminded me of the work in Niedenthal lab (see Langbehn, A. T., Yermol, D. A., Zhao, F., Thorstenson, C. A., & Niedenthal, P. M. (2022). Wearing N95, surgical, and cloth face masks compromises the perception of emotion. Affective Science, 3(1), 105-117.) Yet, I did not find evidence of authors mentioning their group

Action: We broadened our literature review to include additional research on face coverings and emotion recognition, including the suggested reference.

2. Please make sure the statistical reporting, as well as overall article structure is up to APA guidelines and publisher-quality format.
a. The results reporting style is not consistent across two experiments.

Action: We adjusted our analysis to also include the target emotions in both experiments. Along with new approach, we have updated the reporting of the results of those tests and are reported consistently. The additional regression analysis in Experiment 1 is the primary difference between the two experiments and we provided additional information to interpret the results (e.g., simple slopes for interpreting the interaction and confidence intervals for the coefficients).

b. Variables names are changing all the time. I have trouble following. What is the difference between sseit scores and EI. You are reporting both of them.

Action: We changed to using EI except when specifically talking about the measure used throughout the paper.

c. What do you mean by “the slope for the interaction is significant”?? These writings are not acceptable. You need to explain and interpret the results in a professional fashion.

Action: We clarified this is in reference to interaction coefficient from the regression analysis.

3. In the results and analysis section, page 12 – first paragraph, why is the EI x time interaction a violation of assumption? To me, it’s the most interesting findings of the study. The writing does not make any sense

Action: We originally planned to analyze the effect of emotional intelligence as a covariate in an ANCOVA, under the assumption that those with higher emotional intelligence will generally have higher accuracy. That does assume that the influence (slope) of emotional intelligence is similar across conditions (homogeneity of slopes). We tested that assumption before conducting our planned ANCOVA and found that was not the case. We agree that this is an interesting finding, which is why we conducted the follow-up regressions. We included more interpretation of this interaction in the results section (inclusion of the simple slopes of each time condition) and discuss it further in the general discussion. We originally interpreted this finding cautiously given the marginal nature of the regression results.

4. All results had no interpretations.
Action: We originally tried to reduce some of the redundancy between the results and discussions of the individual experiments and then the general discussion. In the revision, we provide more interpretation of the results within each experiment.

5. For experiment 1, I do not see why you are adding the timing as a factor? This was never discussed previously. And why did you choose 1 second in the experiment 2?

Action: Because of the speed of processing facial emotions, we wanted to add some level of ambiguity (or time pressure) for any individual difference like EI to make a meaningful difference. If unlimited time is allowed to sample the stimulus, something as subtle as EI wouldn’t make a meaningful difference. We provided additional clarification leading up to and in Experiment 1.

6. Discussion needs significant improvement.
a. Authors claimed causation between facial masks and emotion recognition accuracy. However, the analysis did not show any causality. Please be careful with the strong claim.

Action: We made significant changes to the general discussion as well. We expanded our analyses in the experiments to test overall recognition accuracy across emotions. In addition, we framed our discussion of the confusion patterns as more descriptive of what we found and compare those patterns to other findings.

b. Again, a list of results reporting, yet no sounding discussion.

Action: Throughout the general discussion we compare our findings to other studies that used similar methods to better fit the current findings into prior research.

**Reviewer 2**
The authors report the results of two experiments investigating the obfuscating effect of face masks on the recognition of facial expressions of emotion. They provide a conceptual replication and extension of prior work on masked emotion recognition by including distractor responses and manipulating the time participants had to view the photographs. Moreover, they examined the relationship between emotional intelligence and emotion recognition accuracy across viewing conditions (i.e., masked vs. fully visible and time constraints). Their studies add to a growing literature suggesting that face masks decrease emotion recognition accuracy.

Major issues:
• The authors discuss three early investigations of the effect of face masks on emotion recognition in their Introduction (Grundmann et al., 2021; Carbon, 2020; and Kastendieck et al., 2022). However, I find that these three studies provide an insufficient discussion of relevant literature to situate the current experiments in the body of work investigating the perception of masked facial expressions. Substantial work has been conducted since this data was collected that is directly relevant to their hypotheses. I suggest the authors conduct a more thorough overview of the literature in the Introduction to better situate this study in the broader literature.

Action: We expanded our review of previous work to include work since 2022.

o Emotional intelligence and recognition of masked facial expressions (the abstract states that no such work has been conducted). For example, Carbon et al. (2022) observed no relationships between self-report and behavioral measures of emotional intelligence and emotion recognition accuracy for masked or fully visible expressions of emotion. Likewise, no relationships were found between measures of emotional intelligence and participants confidence in their judgments. On the other hand, Huc et al. (in press) observed that emotional intelligence was positively related to emotion recognition accuracy for masked faces and especially for masked happy faces.

Action: We have incorporated both of these studies into the manuscript (although we discuss Huc et al. in more depth in the discussion).

o Stimulus presentation timing. At least one study has examined stimulus presentation timing and emotion recognition judgments of masked and fully visible faces (Tsantani et al. 2022) which seems highly relevant to the authors’ work. Shepherd et al. (2022) also examined masked/fully visible facial expression recognition for stimuli shown for 250ms.

Action: We have included additional references to support the rationale behind limiting the view time of the images.

o Emotion recognition accuracy. The authors correctly report (p. 7 para. 2) that “Kastendieck et al. (2022) did not find a decrease in emotion recognition accuracy caused by face masks”. However, this null effect is compared to two studies that observed such an effect which gives the reader the impression that the evidence that face masks impair emotion recognition accuracy is more mixed than the current literature suggests (for example see McCrackin et al., 2023; Rinck et al., 2022; Wong et al., 2022; Gori et al., 2021; Ruba et al., 2020). Other studies (e.g., Langbehn et al., 2022) also specifically examined the impact of masks on facial expressions predominantly expressed on the upper and lower parts of the face which seems relevant to the authors’ discussion.
Action: We now provide a more comprehensive review of more recent research that does make it clear face coverings are consistently found to affect emotion recognition.

• In the Introduction, there is some conceptual confusion between mediation and moderation. The authors report (p. 7, para. 2) that participant age, participant sex, and target age have been found to mediate the relationship between face visibility (masked vs. fully visible) and emotion recognition accuracy. However, to my knowledge, the studies cited only support conclusions about moderation.

Action: Yes, we have clarified this in our revision.

• The Introduction leads the reader to believe that the authors would predict a mask by EI or perhaps even a mask by EI by timing interaction (see the last sentence of p. 8, para. 2). Some clarification as to why the authors did not include these predictions would be helpful.

Action: Leading into Experiment 1, we provided additional rational for the role of time. We initially expected a simple linear relationship between EI and accuracy across conditions because even the 1.5 s condition would be sufficiently difficult (obviously, not the case here).

• More information about why EI is likely to be related to emotion expression recognition is also warranted in the Introduction. For one, results suggestions that high EI individuals show faster reaction times provides little evidence that they will also be more accurate at judging the emotion expressed. Moreover, additional interpretation of results like those of Prajapati (2021) would be helpful to show how they are related to accuracy.

Action: Towards the end of the introduction, we further clarify that the time limitation was meant to leverage the possible processing speed effect of emotional intelligence.

• The confusion matrices included in Experiments 1 and 2 are appealing and provide insight into which emotion expressions were confused with which emotion labels. However, they are descriptive in nature. Therefore, the discussion of these patterns should be limited to descriptive language and terms like significant (e.g., p. 20, para. 1) which give the impression that statistical tests were conducted should be avoided. Alternatively, the authors might consider running such analyses to determine whether masks impair specific emotion expressions significantly more than others.

Action: We clarified the language about the confusion rates to be more descriptive of what we found here and tie it back to other studies that reported confusion matrices. We also reanalyzed our data by including the target emotion as a within-subjects factor. While this does not directly test specific frequencies of erroneous responses across each target expression, but it does allow us to compare correct response rates across emotions and make more comparisons to previous studies that used similar methods. We have still adjusted our language in the general discussion to be more descriptive regarding the rates of confusion.

• The format and writing of the confusion patterns in the General Discussion is difficult to follow, for one, the authors report the percentage of errors here, but it was not immediately clear this was the case (previously they reported the proportion of responses in the tables).

Action: We have reorganized the general discussion in a way we hope is easier to follow. We still discuss the confusion patterns in the context of action units, but we have grouped emotions that showed similar patters (e.g., happiness and surprise) that makes this discussion more cohesive.

o There also appears to be a typo here, “While unmasked fear was most often confused for disgust in the study of Carbon, and fear followed by disgust in both our experiments, in both our mask conditions fear was most often confused for first surprise then disgust.” This appears to say that fear was confused most often for fear in the authors’ experiments.

Action: Thank you for pointing that out. We have addressed this along with the larger reorganization of the general discussion.

• The General Discussion would also benefit from reviewing additional literature.
o The authors focus their discussion on comparing their results to those of Carbon (2020), however, several additional studies have been conducted on the topic.
o Other areas also lack relevant citations (e.g., the authors speculate that religious face coverings may impair emotion recognition a prediction that has been tested [Fischer et al. 2011; Kret et al., 2012]).

Action: Along with the reorganization of the discussion, we have included additional studies to contextualize our results (our application to other contexts were face coverings are used was removed as part of the reorganization of the discussion).

Minor issues:
• In the Results section of Experiment 1 the authors note that a significant EI * time interaction was found for masked faces, however, they do not interpret the interaction until the Discussion. A brief interpretation of this finding would be helpful in the Results section.

Action: We have included the (standardized) simple slopes of emotional intelligence from each time condition to unpack the interaction.

• The introduction seems to indicate that the effect of facial coverings on emotion recognition accuracy and “the potential influence it has on social interactions” would be examined. However, the latter appears to be beyond the scope of the paper (see p.3, para. 1). Some clarification on what is meant by this would help focus the Introduction.

Action: We included the mention of these related social implications as a way to bring a construct like emotional intelligence into our current investigation. We have made our objective clearer in the opening of the paper.

• There appears to be some discrepancy on the number of stimuli the participants in study 1 viewed. P. 10, para. 1 indicates that participants saw 60 experimental trials, however, p. 11, para. 2 indicates they saw 63.

We have clarified this in the method of Experiment 1. We used a total of 62 images, 60 of which were used for experimental trials. One used for an example, and one was used as attention check questions.

• Some clarification is needed in the analysis strategy section for Experiment 2. Did the authors initially conduct an ANCOVA? If so, were all assumptions met?

Action: The assumptions were met in this case, as the time variable was not included and there was no interaction with the mask variable. This was the case with the new analysis as well, and we clarified that in both experiments in the revised manuscript.

• The last sentence of p. 28 appears to be incomplete.

• The manuscript would benefit from some additional editing to ensure clarity of the author’s message. For one, the introduction could be improved by including additional transitions and connections between the studies the authors discuss.

Action (for both of the above): As we reorganized the manuscript, we were more conscientious about flow between sections and clarity.

Reviewer 3

The paper by Libby and Scarince describes two experiments investigating how emotion recognition accuracy is affected by face coverings when faces are presented for a limited viewing time, as well as how individual differences, such as emotional intelligence, modulate this effect. The authors found that recognition accuracy was lower for masked faces compared to unmasked faces in both experiments, with masked expressions of disgust and sadness showing the greatest increase in confusion. Furthermore, emotional intelligence influenced accuracy only when viewing time was particularly restricted, and parts of the face were covered.
Overall, the paper is well-written and clearly presented. I have only a few minor comments that may help improve the manuscript:

- Why were neutral face images not included as target stimuli? They could have served as a useful control condition. Please specify this point.

Action: We were not able to cross masked and unmasked neutral faces across age, gender, and race as well as we were with the other emotions. We also had some practical limitations in the time frame of the study. We had to keep the median completion time at around 15 minutes, and the inclusion of neutral expressions would have pushed us beyond that mark. We provided this clarification in the method of Experiment 1.

- It is unclear why the authors selected these specific viewing times, which are relatively long, instead of other durations. Did they base their choice on previous literature, or was there another criterion for selection? Please clarify.

Action: We added some clarification in the method of Experiment 1. Because this study was run online, we had concerns about the lower limit of how long we could display the images reliably. While testing the procedure, half a second ran well and we used that as the fastest presentation and scaled up from there.

- It is unclear why observation time was considered as a between-subjects factor. Could the authors provide a rationale for this choice?

Action: We ran time as a between-subjects factor because we wanted to prioritize repeated observations across emotions and face coverings. We worried using time as a within-subjects factor would result in fatigue.

- I was wondering why reaction times were not included as dependent variables. Is there a specific reason for this? Additionally, did the authors exclude trials with extremely long or short reaction times from the analysis?

Action: We did not report or analyze RTs in this case due to the large number of possible responses and the participants did not complete the experiments on standardized equipment. These two factors together would increase the noise and make interpreting effects on RTs tenuous. We added this justification in the analysis plan of Experiment 1.

- What was the rationale for introducing the four distractors within the response options? How were these specific distractors selected?

Action: We selected the four distractor responses based on previous work that also included distractors (i.e., Grundman et al., 2021). We removed them in Experiment 2 so we could compare those results to other studies that did not include them. We provided additional clarification for including them in the method of Experiment 1.

Additional Comments to the Academic Editor Not Addressed Above

Journal Requirements:

When submitting your revision, we need you to address these additional requirements.

1. Please ensure that your manuscript meets PLOS ONE's style requirements, including those for file naming.

Action: We have reviewed the style requirements, templates, and file naming requirements.

- We have named the figure files and document files accordingly.
- We reviewed our references to align with Vancouver style.
- We have checked heading and body text font style to match the guidelines
- We have included more information about our statistical analysis including software and practices for interpreting significance.
- We added line numbers throughout the document.

2**.** Please include your full ethics statement in the ‘Methods’ section of your manuscript file. In your statement, please include the full name of the IRB or ethics committee who approved or waived your study, as well as whether or not you obtained informed written or verbal consent. If consent was waived for your study, please include this information in your statement as well.
Action: We have provided additional clarification about the ethics review for this study and given it a section heading. Both experiments in this study were classified as exempt by the TAMUCC IRB (information added in the method section for Experiment 1), and we clarified that the electronic consent was a written form completed at the beginning of the procedure.

3. We note that Figure 1 includes an image of a participant.
Action: The included figure does not include a participant in this study. This is one of the example images of the CK+ image dataset we used, which is permissible use in scientific publishing as long as attribution is provided. Given we are not able to have the person featured in the image to complete the necessary forms, we have included the example in the OSF page for this project and link to it in the manuscript.
