# Supplementary material for: The effect of face masks on confusion of emotional expressions
Source: PLoS One. 2025 Sep 10;20(9):e0330430. doi: 10.1371/journal.pone.0330430 (PMC12422498; doi:10.1371/journal.pone.0330430)
Supplement: Attachment — Submitted filename: LibbyAndScarince_RnR2_Response.docx [file pone.0330430.s003.docx]

**Response to Reviewers**

We would like to begin by thanking Dr. Selvaraj, Dr. Ciricugno, and Reviewer 2 for their time and patience in reviewing our manuscript and providing additional feedback on our work.

Below are our responses to comments, point-by-point, along with the changes made in the manuscript.

**Reviewer 2**

The current version of the manuscript represents a substantial improvement from the previous version. The authors have addressed many of the concerns noted in the initial review. There are, however, a few additional areas that warrant further revision before this manuscript should be accepted for publication.

1.The paragraph beginning on line 66 could benefit from additional editing to make the author's argument clearer.
1a. For example, in Lines 68-71 it is unclear what the “also” refers to.
1b. As reviewer 1 mentioned, Langbehn et al. (2021) is likely relevant here. They tested the prediction that the effect of face masks on emotion intensity judgments would be moderated by whether a given facial expression is predominately displayed using AUs from the upper versus lower part of the face.

Action: We have revised this paragraph to clarify the overall reduction of accuracy when parts of the face are occluded and that some expressions are affected more than others. We also included reference to Langbehn when talking specifically about occluding the lower half of the face for specific emotions (reference 27).

Revision (ln 75): Given that distinct regions of the face are important for interpreting facial expressions, it is not surprising that expression recognition may be impaired if certain parts of the face are not visible [20-25]. While both the eye region and mouth region are significant informants of emotions, Kotsia et al. [17] and Blais et al. [16] determined that occluding the mouth region impairs overall emotion recognition more than occluding other facial regions. Accuracy is further reduced for expressions that have more key AUs in the mouth region than other facial regions, such as disgust [18,26,27]. These findings together indicate that limited visibility of the lower half of the face, such as when a face mask is worn, will impair overall emotion recognition by blocking the observer from necessary diagnostic information. These findings also indicate that face coverings will impair recognition of some emotional expressions more than others, based on the diagnostic information necessary for identification of that expression [27].

2. There are at least two places in the Introduction where research is listed without a clear conceptual link to the current work. Moreover, this research is not reviewed in sufficient detail for the reader to understand what this previous work observed.
2a. Lines 84 – 85: Why are trustworthiness and interpersonal closeness judgments relevant to the current work? If they are relevant, the relevance needs explanation. Moreover, it is unclear whether masks increased, decreased, or had no effect on these judgments.
2b. Lines 105 – 107: The studies reviewed here also seem unrelated to the current work, and it is unclear whether these individual differences moderated the effect of masking on accuracy (or another outcome measure).

Action: The inclusion of these references is to (1) illustrate the broader impacts of face coverings beyond less certainty of what expression someone is showing and (2) help make the connection to why emotional intelligence is relevant to investigate.

We decided to keep most of these references and have added additional context to help them fit in the respective sections of the paper.

Revision (ln 92): In addition, face masks impact recognition differentially based on the AUs covered by face masks. For example, emotions that are expressed more with the eyes, such as anger, are less affected by face masks than other expressions [38]. Research on broader effects on interpersonal perception have found wearing facial coverings lowers trustworthiness [33, 39] and reduce feelings of interpersonal closeness [39,40].

Revision (ln 117): Research on broader effects on social perception have found wearing facial coverings lowers feelings of trustworthiness toward the target [33, 39] and reduces feelings of interpersonal closeness [39,40].

Revision (ln 137): Individual differences in mental wellness have also been investigated. Men with high perceived stress have been found to take longer to identify masked happiness than men with low or moderate stress while the inverse was found for women [45].

3. Lines 119 – 123:
3a. The null effect of self-reported EI on accuracy could potentially be explained by low internal reliability. However, the null relationship between the ability-based EI measure and accuracy could not be explained by this.
3b. The abstract also highlights the mixed findings between EI and emotion recognition accuracy. However, the introduction only reviews one study indicating that people with high EI respond faster when categorizing emotion expressions and one study that found no relationship between EI and recognition accuracy. Huc et al. (2025) is not mentioned until the Discussion, however, it seems highly relevant here.

Action: We added Huc et al. (2025) to the introduction (and corrected the citation) to the following the summary of Carbon et al. (2022). We then contrasted the two methodologies to point out that the null effect Carbon reported could be due to the unrestricted time targets were visible, limiting the potential effect of EI.

Revision: Recently, Huc et al. [53] investigated of how several psychological individual differences—including perceived stress, loneliness, psychological disorders, and EI—affect expression recognition when faces are covered. Unlike Carbon’s [30] procedure, targets were presented for only 450 ms. Their results indicated that EI was a positive predictor for recognition accuracy, particularly for happy expressions. The difference in target presentation duration might account for the discrepancy between Huc et al.’s positive findings and Carbon’s null results. EI may only reliably predict expression recognition when there is high ambiguity. When an expression is unobstructed and visible for an extended time, both high- and low-EI individuals can effectively process the expression. The less clear the signal is, like when part of the face is covered and the expression is fleeting, the more individual differences in processing emotional signals might affect recognition.

4. The term “Lure” responses is used differently across the paper, which makes it confusing. Sometimes it is used to refer to distractor responses (those that are never the correct answer [e.g., Lines 334-337, 409-411]); it also appears to refer to all potential responses (i.e., Lines 307-309).

Action: "Lure" is meant to only include response options for expressions that were not in the image set.

Revision (ln 283): This question included ten response options including six basic emotions (anger, fear, surprise, sadness, disgust, and happiness) as well as four lures that were never the correct response (neutral, tiredness, annoyance, and excitement) [39].

Revision (ln 321): Second, we removed lure responses to the emotion recognition task, as some options in Experiment 1 could reasonably be conflated with one another based on subtle differences and interpretation of expressions.

5. For Lines 309-312, what evidence do you have that the neutral response functioned as a decision default in Experiment 1? Participants rarely used the neutral response for unmasked targets but occasionally used it for masked targets. As written, this argument does not seem compelling.

Action: This was based on the descriptive trends in the confusion matrix that lures were frequently selected in the masked condition and this was true for neutral responses for most emotions. We revised this paragraph to include another example of conflated expressions and changed the language around the neutral response to reflect our descriptive interpretation of how the lure responses affected participant responses.

Revision (ln 420): For instance, annoyance was frequently conflated with anger in both masked and unmasked conditions. Furthermore, neutral could be used as a functional “default” response when there are not strong signals for an expression given its higher response rate in the masked conditions for several target emotions.

6. The results of Experiment 2 do not seem to fully support the conclusion in lines 392-394. Experiment 2 did not find “increased confusion rates” for all emotion expressions (Figure 4). For example, anger showed no difference in accuracy, no change in confusion for happiness, and, if anything, reduced confusion with disgust and sadness when masked. Fear also showed a large decrease in confusion with disgust when masked compared to when unmasked.

Action: We adjusted language to specify this interpretation is regarding the higher diversity in error response in the masked condition.

Revision (ln 518): Regarding confusion rate patterns between mask conditions, we found more varied error responses across most emotions in the masked condition, although anger, fear, and happiness were more robust against confusion in general.

7. The results of Experiment 1 do not seem to support the conclusions in Lines 395-396. Experiment 1 found a significant simple effect of EI on masked emotion recognition accuracy when targets were presented for 1 second. This was similar to the simple effect of EI when the targets were presented for 0.5 seconds.

Action: We included additional speculation on the reasoning for not replicating the positive relationship for the 1 s view time from Experiment 1 in Experiment 2.

Revision (ln 523): In this experiment, we did not find a relationship between EI and recognition accuracy in general or for either mask condition. Although the results of Experiment 1 suggested a relationship between accuracy and EI would be found in the masked condition, the removal of the lure responses made the recognition task easier, thus reducing the effect of EI.

8. Lines 426-428 would benefit from additional editing to make it clear which expressions are being confused with which expressions.

Action: We provided clarification that the confusion patterns in that section are regarding happiness and surprise.

Revision (ln 617): Confusing happiness and surprise with a neutral expression [29,30] may be due to insufficient visible diagnostic information or lack of response confidence. The shared AUs between happiness and surprise also account for how frequently they are confused for each other [55].

9. There is an error in Lines 436-437. This sentence currently says that AU 4 is a component of sadness, which was a common error for both sadness and disgust.

Action: We corrected that the expression sadness and disgust were being compared to anger.

Revision (ln 628): AU 4 is also a component of anger, which was a common error for both sadness and disgust targets.

10. Lines 441-443 and Lines 452-453 appear to have conflicting citations. Proverbio and Cerri (2022) is cited as showing both a limited effect of masking on the recognition of anger and no effect (they found no effect). Leitner et al (2022) is also cited for both claims (they found no effect). The remaining citations in this paragraph need to be reviewed for accuracy.

Action: We reviewed the citations in this section (and across the paper). We separated the list of citations in this section to better align with the parts of the statement they support.

Revision (ln 633): The low recognition for fear is also consistent with previous studies that have found lower overall accuracy for fear [42, 59] and limited effects of masks [36, 42] or no effect [29-31, 60]. The AUs characteristic of anger around the eyes (AUs 4, 5, and 7) are all shared with at least two other emotions [19]. This may account for the relatively low accuracy in recognizing anger. The high rates of confusion with disgust may be attributed to the shared AUs 4 and 7, while the raised upper lip (AU 9) important for identifying disgust is obscured in masked conditions. Similarly, sadness shares AU 4 with anger, one of only two visible indicators of sadness when images are masked while AU 4 and 5 are shared between anger and fear, leaving only one of three visible indicators of fear. This suggests that confusion patterns for masked anger relate to lack of information for other emotions more than for lack of identifiers of anger. The inconsistent findings for anger between the experiments reported here reflect the inconsistency from previous studies. Some investigations have found effects of masks on recognition of anger [29, 31, 32, 42, 60] while others have found no difference [40, 41, 59]. Indeed, Mikayzaki et al. [35] also found mixed effects between the two experiments they conducted.

11. Lines 484-486: Carbon (2022) did not find a relationship between EI (ability or self-reported) and emotion recognition accuracy. If the authors believe that Carbon’s results were found in error, they need to explain why this is the case. Moreover, Huc et al. (2025) found a positive relationship between self-reported EI and accuracy.

Action: Action: We were not necessarily trying to make the case that the results are in error, but that the effect of EI is contextual and sensitive to how it is defined and, thus, measured. We have clarified this in the final paragraph of our limitations and future directions section.

Revision: Because of the sensitivity of this effect, the way EI is assessed might affect how it can be detected given the conditions of an experiment. In the current investigation we used a self-report measure of EI, as did Huc et al. [53], rather than an ability assessment, like the Mayer-Salovey-Caruso Emotional Intelligence Test [67] used by Carbon et al. [30]. Measuring EI with an ability assessment might be also effective at identifying individual differences in emotion processing under conditions of ambiguity.

12. Huc et al. (2023) did not include EI in their study (Lines 456-457, 459-462). The correct reference is Huc et al. (2025).
a. Huc, M., Bush, K., Berrigan, L., Cox, S., Jaworska, N. (2025). The influence of emotional intelligence on facial expression processing in males and females with and without psychiatric illnesses. Personality and Individual Differences, 237, 113040. <https://doi.org/10.1016/j.paid.2025.113040>.

Action: We have included the correct reference.

13. Typographic Errors
13a. Line 122: et al.
13b. Line 180: “was” should be “were”
13c. Line 352: missing “with”.
13d. Line 476: missing “and” before “still”.

Action: We have revised the identified errors and made additional minor changes throughout the manuscript.

**Academic Editor / Journal**

If the reviewer comments include a recommendation to cite specific previously published works, please review and evaluate these publications to determine whether they are relevant and should be cited. There is no requirement to cite these works unless the editor has indicated otherwise.

Action: We have included the recommended references (see below).

Please review your reference list to ensure that it is complete and correct. If you have cited papers that have been retracted, please include the rationale for doing so in the manuscript text, or remove these references and replace them with relevant current references. Any changes to the reference list should be mentioned in the rebuttal letter that accompanies your revised manuscript. If you need to cite a retracted article, indicate the article’s retracted status in the References list and also include a citation and full reference for the retraction notice.

Action: We included one reference that was erroneously excluded in the previously submission (Huc et al., 2025; *Frontiers in Psychology*).

We also removed one reference that is no longer needed following some of our revisions (Taylor & Asmundson, 2021; *PLOS One*).

No references have been retracted.

While revising your submission, please upload your figure files to the Preflight Analysis and Conversion Engine (PACE) digital diagnostic tool, [https://pacev2.apexcovantage.com/](https://nam12.safelinks.protection.outlook.com/?url=https%3A%2F%2Ftrack.editorialmanager.com%2FCL0%2Fhttps%3A%252F%252Fpacev2.apexcovantage.com%252F%2F1%2F010f01981e0e2f43-5d948787-9b12-4bbe-a663-a01eed09d6eb-000000%2FZ3WHmH6trkiGiV2J7JjocBhk9MXgsf8RSP01-W0ZLTI%3D220&data=05%7C02%7Ccollin.scarince%40tamucc.edu%7C4fdd157e6b2a44e1acb208ddc60c0220%7C34cbfaf167a64781a9ca514eb2550b66%7C0%7C0%7C638884477368476049%7CUnknown%7CTWFpbGZsb3d8eyJFbXB0eU1hcGkiOnRydWUsIlYiOiIwLjAuMDAwMCIsIlAiOiJXaW4zMiIsIkFOIjoiTWFpbCIsIldUIjoyfQ%3D%3D%7C0%7C%7C%7C&sdata=bQTBDj2%2FC7%2BtK6kcE74Y%2BsSgY7vrXRmvyg5A45bjaE0%3D&reserved=0). PACE helps ensure that figures meet PLOS requirements. To use PACE, you must first register as a user. Registration is free. Then, login and navigate to the UPLOAD tab, where you will find detailed instructions on how to use the tool. If you encounter any issues or have any questions when using PACE, please email PLOS at [figures@plos.org](mailto:figures@plos.org). Please note that Supporting Information files do not need this step.

Action: Updated figures were run through PACE and uploaded with this submission.
